# *COL5A1* rs12722 polymorphism is not associated with passive muscle stiffness and sports-related muscle injury in Japanese athletes

**DOI:** 10.1186/s12881-019-0928-2

**Published:** 2019-12-02

**Authors:** Eri Miyamoto-Mikami, Naokazu Miyamoto, Hiroshi Kumagai, Kosuke Hirata, Naoki Kikuchi, Hirofumi Zempo, Noriko Kimura, Nobuhiro Kamiya, Hiroaki Kanehisa, Hisashi Naito, Noriyuki Fuku

**Affiliations:** 1 Institute of Health and Sports Science & Medicine, Juntendo University, 1-1 Hiraka-gakuendai, Inzai City, Chiba, 270-1695 Japan; 2 Juntendo Advanced Research Institute for Health Science, Juntendo University, Tokyo, Japan; 3 Graduate School of Health and Sports Science, Juntendo University, Chiba, Japan; 4 Research Fellow of Japanese Society for the Promotion of Science, Tokyo, Japan; 5 Graduate School of Engineering and Science, Shibaura Institute of Technology, Saitama, Japan; 6 Department of Training Science, Nippon Sport Science University, Tokyo, Japan; 7 Faculty of Health and Nutrition, Tokyo Seiei College, Tokyo, Japan; 8 Graduate School of Sport and Exercise Sciences, Osaka University of Health and Sport Sciences, Osaka, Japan; 9 Faculty of Budo and Sport Studies, Tenri University, Nara, Japan; 10 Faculty of Sport and Health Science, Ritsumeikan University, Shiga, Japan

**Keywords:** Muscle injury, Muscle stiffness, Joint flexibility, Single nucleotide polymorphism, Type V collagen

## Abstract

**Background:**

Poor joint flexibility has been repeatedly proposed as a risk factor for muscle injury. The C-to-T polymorphism (rs12722) in the 3′-untranslated region of the collagen type V α1 chain gene (*COL5A1*) is reportedly associated with joint flexibility. Flexibility of a normal joint is largely determined by passive muscle stiffness, which is influenced by intramuscular collagenous connective tissues including type V collagen. The present study aimed to test the hypothesis that the *COL5A1* rs12722 polymorphism influences joint flexibility via passive muscle stiffness, and is accordingly associated with the incidence of muscle injury.

**Methods:**

In Study 1, we examined whether the rs12722 polymorphism is associated with joint flexibility and passive muscle stiffness in 363 healthy young adults. Joint flexibility was evaluated by passive straight-leg-raise and sit-and-reach tests, and passive muscle stiffness was measured using ultrasound shear wave elastography. In Study 2, the association of the rs12722 polymorphism with sports-related muscle injury was assessed in 1559 Japanese athletes. Muscle injury history and severity were assessed by a questionnaire. In both Study 1 and Study 2, the rs12722 C-to-T polymorphism in the *COL5A1* was determined using the TaqMan SNP Genotyping Assay.

**Results:**

Study 1 revealed that the rs12722 polymorphism had no significant effect on range of motion in passive straight-leg-raise and sit-and-reach tests. Furthermore, there was no significant difference in passive muscle stiffness of the hamstring among the rs12722 genotypes. In Study 2, rs12722 genotype frequencies did not differ between the muscle injury and no muscle injury groups. Moreover, no association was observed between rs12722 polymorphism and severity of muscle injury.

**Conclusions:**

The present study does not support the view that *COL5A1* rs12722 polymorphism has a role as a risk factor for sports-related muscle injury, or that it is a determinant for passive muscle stiffness in a Japanese population.

## Background

Despite preventive efforts, muscle injury is increasingly common in sports involving sprinting and jumping [1]. Muscle injury results in considerable losses such as missed training time and unavailability for competition and thereby negatively influences the athletes’ sport success. Thus, it is essential to elucidate the etiology of sports-related muscle injury in order to develop a more effective prevention modality.

Poor joint flexibility has been well documented as a modifiable risk factor for muscle injury. A prospective study [2] indicated a direct link between pre-season joint flexibility and muscle injury in soccer players; with injured players having a significantly smaller joint range of motion (ROM) measured pre-season than uninjured players. Joint flexibility is influenced by environmental factors such as stretching [3, 4] and resistance training [5]. In contrast, a meta-analysis showed that genetic factors explain 50% of joint flexibility variance [6]. Thus, the influences of genetic and environmental factors on joint flexibility are comparable.

Other than the commonly accepted extrinsic factor, genetic factors play an important role in the risk of muscle injury [7, 8]. The collagen type V α1 chain gene (*COL5A1*) has been proposed as a candidate gene whose variants affect not only muscle injury per se but also passive muscle stiffness and joint flexibility [9]. Several studies examining the association of the C-to-T polymorphism (rs12722) in the 3′-untranslated region (UTR) of the *COL5A1* with ROM in sit-and-reach (SR) or passive straight-leg-raise (PSLR) tests [10–12] suggest that this polymorphism could be a factor affecting ROM. Although the ROMs in SR and PSLR tests are multifactorial, passive muscle stiffness has been proposed to be a major contributor to the ROM of normal joints [13, 14]. Passive muscle stiffness is influenced by intramuscular collagenous connective tissues, such as perimysium and endomysium [15], which contain type V collagen [16]. *COL5A1* is reportedly expressed in skeletal muscle tissue [17]. Given this, it is assumed that the *COL5A1* rs12722 polymorphism influences ROM via passive muscle stiffness, and is accordingly associated with muscle injury. To date, two studies suggest that the *COL5A1* rs12722 polymorphism is associated with severity of muscle injury in Caucasian professional soccer players [18, 19]. However, the association of the *COL5A1* rs12722 polymorphism with passive muscle stiffness and incidence of muscle injury remains unclear.

The C-to-T rs12722 polymorphism is reported to be associated with altered stability of *COL5A1* mRNA [20]. The *COL5A1* 3′-UTR with the rs12722 polymorphism T allele exhibited enhanced mRNA stability compared to the 3′-UTR of the C allele, suggesting that more type V collagen α1 chain is synthesized from the T allele. Although type V collagen is a quantitatively minor fraction fibrillar collagen, it plays a critical role in the regulation of collagen fibril assembly and is an important structural component of tendons, ligaments, and other connective tissues [9]. As suggested by Collins and Posthumus [9], it is reasonable to assume that the T allele of the *COL5A1* rs12722 polymorphism leads to higher type V collagen production and accordingly alters collagen fibril architecture, resulting in changes in the mechanical properties of the connective tissues. Based on these considerations, we hypothesized that the T allele of the *COL5A1* rs12722 polymorphism is associated with high muscle stiffness, and accordingly poor joint flexibility and a high incidence of muscle injury. To test this hypothesis, we examined the association of *COL5A1* rs12722 polymorphism with passive muscle stiffness, ROM, and muscle injury.

## Methods

### Study design

We performed two studies to fulfill the goals of this paper. The first study (Study 1) was designed to examine the association between *COL5A1* rs12722 polymorphism and joint ROM and passive muscle stiffness. In the second study (Study 2), we investigated whether *COL5A1* rs12722 polymorphism was associated with sports-related muscle injury. Study 2 was a part of the Japanese Human Athlome Project (J-HAP) in “Athlome Project Consortium” [21].

### Study 1

A total of 363 healthy young adults (men, *n* = 231; women, *n* = 132) participated in Study 1. None of the participants had apparent neurological, orthopedic, or neuromuscular problems. None of the participants reported muscle soreness or fatigue in the lower limbs at the time of testing. Written consent was obtained from each participant. The procedure was approved by the ethics committee of the Juntendo University and National Institute of Fitness and Sports in Kanoya, and performed in accordance with the Declaration of Helsinki.

The PSLR (right leg) test, SR test, and passive muscle stiffness measurements were performed on each participant in a randomized order. Room temperature for all measurements was kept at 24 ± 2 °C to minimize potential effects of temperature-induced changes in tissue mechanical properties and participants’ sensation to muscle stretch. Prior to the measurements the participants were not allowed to warm-up or stretch. The procedures have been described in detail previously [14]. Briefly, in the PSLR test, participants lay supine with their legs straight on an examining bed. The pelvis and non-testing (left) leg were secured to the bed. The right hip joint was passively flexed, with the knee straight, by an examiner until each participant felt pain in the hamstring. The PSLR ROM (i.e., hip flexion angle from the resting position) was measured using a digital inclinometer (MLT-100, Sakai Medical, Japan) attached to the right shank. In the SR test, participants sat on the floor with their head, back, and hip against a wall, knee fully extended, and soles of the foot positioned flat against an SR box (T.K.K.5111, Takei Scientific Instruments, Japan). They were then asked to bend forward slowly and reach forward as far as possible while keeping the knees extended and slid their hands along a digital measuring scale which was placed on the box to measure SR ROM.

In accordance with a previous study, passive shear modulus (a measure of stiffness, expressed in kPa) of the biceps femoris long head (BF), semitendinosus (ST), and semimembranosus (SM) of the right leg were measured using an ultrasound shear wave elastography scanner (Aixplorer, Supersonic Imagine, France). During the measurement of passive muscle stiffness, participants sat on a bench with their hip flexed 70° and the right knee fully extended. This hip joint angle was chosen based on a recent study [14], which aimed to define an angle where the hamstring could be stretched to a tensioned state without pain for all participants and the shear modulus could be quantified at a given joint angle in all participants. An ultrasound linear probe was positioned at 50% level of the thigh length (the distance between the greater trochanter and the lateral epicondyle of the femur). For each muscle, the probe orientation was adjusted to visualize the fascicles within the B-mode image. Care was taken not to press and deform the muscles while scanning. The subjects were instructed to fully relax the leg throughout the measurements. The images were acquired after ensuring a stable color distribution of shear modulus mapping for a few seconds. The measurements were performed three times for each muscle. To evaluate the stiffness of the overall hamstring, the shear modulus of three muscles were averaged. All measurements and analyses of the elastographic data were performed by experienced examiners (>3 years of practice). For each variable (i.e., SR test, PSLR test, and passive muscle stiffness), the average values of three measurements were used for subsequent analyses. All participants then completed a questionnaire that included information on regular stretching of the hamstring.

### Study 2

Participants of study 2 were Japanese athletes of various sports (not limited to a specific discipline), recruited from March 2015 to November 2017. A total of 2181 participants were recruited. Written consent was obtained from each participant. The procedure was approved by the Ethics Committees of Juntendo University, Nippon Sport Science University, Tenri University, and the National Institute of Fitness and Sports in Kanoya, and performed in accordance with the Declaration of Helsinki.

In J-HAP, the history of sports-related injuries was assessed by questionnaire [8]. In the present paper, we focused on non-contact muscle injury diagnosed by medical practitioners. The number of days that elapsed from the date of injury to the date of the athlete’s return to usual training was asked to evaluate the severity of injury via the questionnaire. Injury severity was categorized into one of four levels based on the number of days until return: minimal (1–3 days), mild (4–7 days), moderate (8–28 days), and severe (>28 days) [22]. If a participant had a history of multiple muscle injuries, data on the most severe injury was used. In addition, precise information on the primary sport, playing years, and competition level was obtained using the questionnaire. Participants who had [1] no Japanese ancestry [2], missing or invalid questionnaire data, or [3] less than 3 years playing experience in their primary sport were excluded. The final sample size in the analyses of history of muscle injury and severity of muscle injury were 1559 and 186, respectively.

### Genotyping analysis

Total DNA was isolated from the saliva of all participants in both Study 1 and 2 with an Oragene® DNA Collection Kit (DNA Genotek, ON, Canada) and quantified using a NanoDrop 8000 UV-Vis Spectrophotometer (Thermo Fisher Scientific, DE, USA) or Eppendorf Bio Photometer Plus (Eppendorf, Tokyo, Japan). DNA samples were stored at 4 °C until use. The samples were analyzed for the rs12722 polymorphism in the 3′-UTR of *COL5A1* using a TaqMan SNP Genotyping Assay (Assay ID: C____370252_20) and LightCycler® 480 System (Roche Molecular Systems, Mannheim, Germany) or StepOne™ Real-Time PCR System (Thermo Fisher Scientific). Five microliters of the genotyping mixture contained 2.5 μL TaqMan® GTXpressTM Master Mix (2×) or TaqMan® Universal Master Mix II (2×), 0.0625 μL TaqMan® SNP Genotyping Assay mix (40×), 1.4375 μL sterilized water, and 1 μL genomic DNA (10 ng/μL). Two to four negative controls were included on each plate. Genotypes were called based on TaqMan® assays results using LightCycler® 480 SW (version 1.5, Roche Molecular Systems) or StepOne™ software (version 2.3, Thermo Fisher Scientific). Three hundred eighty randomly selected samples were genotyped in duplicate for the rs12722 polymorphism, and we confirmed that the genotyping results perfectly agreed between duplicates.

### Statistical analysis

Data are expressed as mean ± standard deviation (SD). Statistical significance was set at *P* < 0.05. Statistical analyses were performed using JMP Pro version 12 (SAS Institute, USA). The Hardy-Weinberg equilibrium of the rs12722 polymorphism was assessed using χ^2^ test. For the data of study 1, comparisons between two groups (sex, regular stretching habit, genotype [T-dominant and T-recessive models]) were conducted using χ^2^ test or unpaired Student’s t-test as appropriate. For comparisons under an additive model, the Cochran-Amitage trend test or Spearman correlation test was used as appropriate. Additionally, in order to examine whether the genotypes are associated with the phenotype variables independently of sex and regular stretching, analysis of covariance (ANCOVA) and multiple regression analysis were employed for T-dominant and T-recessive models and for an additive model, respectively. For the data of study 2, comparisons between the muscle injury and no muscle injury groups were conducted by χ^2^ test or unpaired Student’s t-test. Logistic regression analysis was applied to investigate the associations between rs12722 polymorphism and history of muscle injury with adjustment for playing years and main sport (track & field or the others). Odds ratios (OR) and 95% confidence intervals (CI) were calculated under the T-dominant genetic model. Association of the rs12722 polymorphism with severity of muscle injury was assessed by ordinal regression analysis. When the sample size of the TT genotype of the rs12722 polymorphism was less than five, statistical analyses were not conducted for T-recessive and additive models. Using the data of our previous study [8], we calculated the necessary samples size to detect the expected difference in passive muscle stiffness between the T allele carriers and CC genotype carriers (α = 0.05, power = 0.8, difference in group means = 3.5, within group SD = 7.4, and the ratio of CC genotype carriers to T allele carriers = 2.3) and to detect the association between the T allele of the rs12722 polymorphism and a history of muscle injury with an OR of 2.0 (α = 0.05, power = 0.8, probability of T allele carriers = 0.317, and the ratio of control to case subjects = 8.9). The critical sample sizes were estimated to be 168 and 733, respectively.

## Results

The *COL5A1* rs12722 genotype frequencies in studies 1 and 2 did not deviate from Hardy-Weinberg equilibrium (*P* = 0.975 and *P* = 0.183).

### Study 1

Table 1 shows descriptive data on the characteristics of the participants in study 1. There were significant sex differences in height, body mass, PSLR ROM, SR ROM, and muscle shear modulus of the ST and SM (Table 1). When the participants were divided by stretching habit, participants conducting regular stretching showed significantly higher PSLR (84.4 ± 17.5 degree vs. 75.6 ± 14.9 degree, *P* < 0.001) and SR (11.5 ± 9.0 cm vs. 6.5 ± 10.1 cm, *P* < 0.001) ROMs and lower shear modulus of SM (38.6 ± 15.3 kPa vs. 46.4 ± 19.7 kPa, P < 0.001) than those without a stretching habit. The χ^2^ test revealed no significant difference in sex ratio and regular stretching among those with the various rs12722 genotypes (Table 2). PSLR and SR ROMs did not differ among the genotypes (Table 2). Similarly, no significant effect of genotype on PSLR and SR ROMs was observed after adjustment for sex ratio and regular stretching (Table 2).

**Table 1:** Participant characteristics in Study 1 (*n* = 363)

	All	Men	Women	*P* value Men vs. Women
n	363	231	132	
Age, year	20.5 ± 2.0	20.7 ± 2.0	20.2 ± 1.9	**0.043**
Height, cm	169.3 ± 9.0	174.0 ± 6.6	161.2 ± 6.5	**<0.001**
Body mass, kg	64.2 ± 11.6	68.4 ± 11.5	56.8 ± 7.2	**<0.001**
PSLR ROM, degree	82.3 ± 17.3	77.1 ± 14.4	91.5 ± 18.1	**<0.001**
SR ROM, cm	10.43 ± 9.5	8.3 ± 9.6	13.9 ± 8.2	**<0.001**
Shear modulus (kPa)^a^				
Biceps femoris	19.9 ± 7.0	19.8 ± 7.3	20.1 ± 6.4	0.652
Semitendinosus	25.8 ± 8.6	27.3 ± 9.1	22.9 ± 6.4	**<0.001**
Semimembranosus	40.4 ± 16.7	42.5 ± 17.4	36.2 ± 14.5	**0.001**
Stretch, % yes	78.2	73.9	81.1	0.123

Values are presented as the mean ± SD unless noted otherwise. Bold emphasis: *P* < 0.05

^a^Data are available 353, 353, 331 participants for the biceps femoris, semitendinosus, and semimembranosus, respectively

**Table 2:** Participant characteristics by *COL5A1* genotype in Study 1 (*n* = 363)

	CC	TC	TT	Unadjusted/adjusted^a^ *P* value		
				T-dominant (TT + TC vs. CC)	T-recessive (TT vs. TC + CC)	Additive (TT vs. TC vs. CC)
n	253	100	10			
Sex, % men	60.8	71.0	60.0	0.097	0.809	0.169
Age, year	20.5 ± 1.9	20.7 ± 2.3	19.5 ± 1.5	0.597	0.105	0.828
Height, cm	169.0 ± 9.2	170.6 ± 8.7	166.4 ± 7.7	0.227	0.289	0.320
Body mass, kg	63.5 ± 10.6	66.2 ± 13.8	61.3 ± 7.2	0.084	0.420	0.404
PSLR ROM, degree	83.4 ± 18.0	81.0 ± 17.2	83.4 ± 12.2	0.274/0.761	0.907/0.986	0.528/0.786
SR ROM, cm	10.4 ± 9.3	9.8 ± 10.2	13.4 ± 7.1	0.781/0.645	0.298/0.323	0.993/0.477
Stretch, % yes	78.2	72.0	80.0	0.261	0.792	0.368

Values are presented as the mean ± SD unless noted otherwise

^a^For PSLR and SR, adjusted for sex and regular stretching

**Fig. 1:**
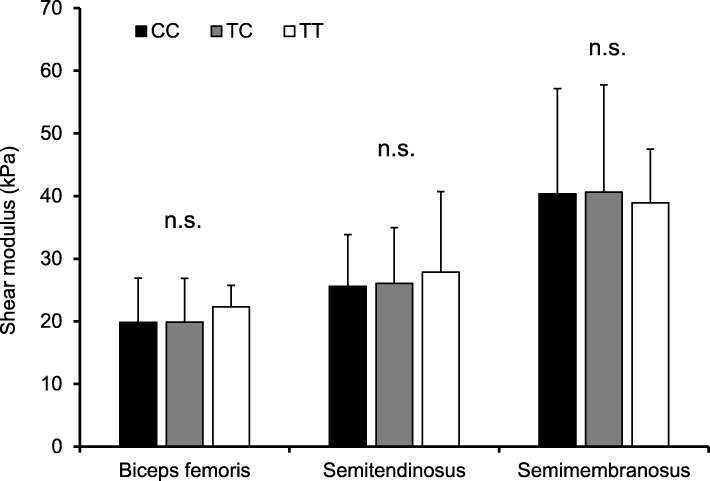
Stiffness of the biceps femoris long head (*n* = 353), semitendinosus (*n* = 353), and semimembranosus (*n* = 331) muscles by the *COL5A1* rs12722 genotype. Data are expressed as the mean ± SD

Figure 1 shows the muscle shear modulus for each rs12722 genotype. There was no significant difference in the shear modulus of all three muscles under three genetic models, regardless of the adjustment for sex ratio and regular stretching (unadjusted: *P* ≥ 0.268, adjusted: *P* ≥ 0.276, Fig. 1). When the shear moduli of the overall hamstring were compared among the rs12722 genotypes, no significant difference was observed either before (T-dominant: *P* = 0.650, T-recessive: *P* = 0.747, Additive: *P* = 0.618) or after adjustment for sex ratio and regular stretching (T-dominant: *P* = 0.958, T-recessive: *P* = 0.835, Additive: *P* = 0.987). In addition, when the participants were divided by sex, genotype had no significant effect on PSLR/SR ROMs or the shear modulus for any of the three muscles in either sex group (Additional file 1: Table S1).

### Study 2

Table 3 shows the participant characteristics in study 2. There were no significant differences in sex ratio, age, height, body mass, and competitive level between the muscle injury and no muscle injury groups. The muscle injury group had fewer playing years and a higher proportion of track and field athletes than the no muscle injury group. Logistic regression analyses of the rs12722 polymorphism (as a whole or for males and females separately) revealed that the genotype frequencies did not differ between the muscle injury and no muscle injury groups (Table 4).

**Table 3:** Characteristics of subjects in muscle injury and no muscle injury group in Study 2

	Muscle injury (*n* = 190)	No muscle injury (*n* = 1369)	*P* value
Sex			0.457
Men, n (%)	134 (70.5)	929 (67.9)	
Women, n (%)	56 (29.5)	440 (32.1)	
Age, years	20.1 ± 1.7	20.5 ± 2.8	0.096
Height, cm	169.9 ± 7.9	169.5 ± 8.3	0.552
Body mass, kg	64.4 ± 10.4	63.4 ± 10.1	0.229
Playing years, years	10.4 ± 3.8	11.2 ± 3.9	**0.008**
Competitive level			0.168
International n (%)	17 (9.0)	153 (11.2)	
National n (%)	112 (59.0)	756 (55.2)	
Regional n (%)	30 (15.8)	286 (20.9)	
Other n (%)	31 (16.3)	174 (12.7)	
Main sport			**<0.001**
Track & field	91 (47.9)	341 (24.9)	
Soccer	62 (32.6)	599 (43.8)	
Other	37 (19.5)	429 (31.3)	

Values are presented as the mean ± SD unless noted otherwise. Bold emphasis: *P* < 0.05

**Table 4:** Associations of *COL5A1* rs12722 genotype with muscle injury

Genotype	n (%)		Dominant TT + TC vs. CC	
	Muscle injury	No muscle injury	OR [95% CI]	*P* value
All				
CC	135 (71.1)	935 (68.6)	0.87 [0.62–1.22]	0.428
TC	52 (27.4)	400 (29.2)		
TT	3 (1.6)	34 (2.5)		
Men				
CC	95 (70.9)	628 (67.6)	0.88 [0.57–1.30]	0.513
TC	37 (27.6)	277 (29.8)		
TT	2 (1.5)	24 (2.6)		
Women				
CC	40 (71.4)	307 (69.8)	0.86 [0.45–1.59]	0.642
TC	15 (26.8)	123 (28.0)		
TT	1 (1.8)	10 (2.3)		

*CI* Confidence intervals, *OR* Odds ratio. Values were adjusted by playing years and main sport

CC genotype was considered as the reference (OR = 1.00)

**Fig. 2:**
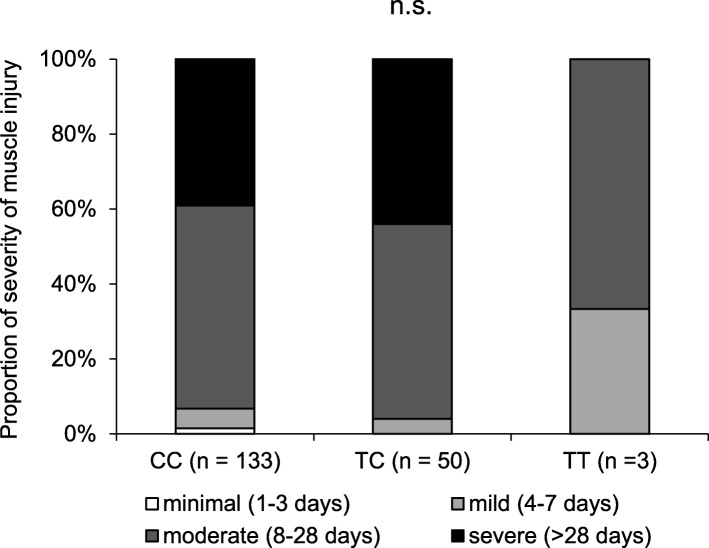
Proportion of severity of muscle injury by the *COL5A1* rs12722 genotype

Figure 2 shows the distribution of muscle injury severity in each rs12722 genotype. There was no significant difference in the distribution of injury severity between participants with TT + TC genotype and those with the CC genotype at the rs12722 polymorphism (*P* = 0.717).

## Discussion

The main finding obtained here is that the *COL5A1* rs12722 polymorphism is not associated with joint ROM, passive muscle stiffness, or a history of muscle injury. These results rule out our hypothesis and suggest that at least in a Japanese population, passive muscle stiffness and muscle injury risk is not affected by rs12722 polymorphism.

In recent years, a growing number of studies have paid attention to genetic susceptibilities to musculoskeletal soft tissue injuries such as tendon and ligament injuries [23–29]. However, limited evidence is available on the genetic component of muscle injury. In the present paper, we focused on the *COL5A1* rs12722 polymorphism and hypothesized that the *COL5A1* rs12722 T allele would be associated with greater susceptibility to muscle injury. To test this hypothesis, we conducted a case-control association analysis in 1559 Japanese athletes, which was much larger than the sample size required to detect the potential association with enough statistical power (*n* = 733). However, contrary to our hypothesis, no association was found between the *COL5A1* rs12722 polymorphism and muscle injury. Additionally, previous studies reported that the *COL5A1* rs12722 polymorphism was associated with the severity of muscle injury in professional soccer players [18, 19] whereas no association was found between the *COL5A1* rs12722 polymorphism and the injury severity in the present study. Participants in the present study were athletes from various sports (not limited to a specific discipline) and with various competitive levels. The polymorphism was not associated with muscle injury history/severity even when examining each sports discipline (i.e., soccer, track & field, etc.) and competitive level (i.e., international, national, and regional) individually (data not shown). The reasons for the discrepancy between the hypothesis and the results are unclear at this time, but we will discuss some possible explanations.

Assuming that intramuscular connective tissues such as the perimysium and endomysium contain type V collagen [16] and contribute largely to passive muscle stiffness [15], we expected that passive muscle stiffness would be influenced by the rs12722 polymorphism. However, the present study found no association between the rs12722 polymorphism and passive muscle stiffness. Passive muscle stiffness is reportedly influenced by not only the connective tissue but also the presence of titin, a giant sarcomeric protein with a wide range of functions [30]. In cardiac muscle, at shorter sarcomere lengths, passive tension development of muscle fibers mainly depends on titin, while at longer sarcomere lengths it is determined by extracellular collagen fibers [31]. Muscle shear modulus measured in a sitting position in the present study were approximately 54% (BF), 43% (ST), and 31% (SM) of those measured at maximal ROM [32]. Taking these aspects into account, it seems that the contribution of intramuscular connective tissues to passive muscle stiffness might be small in the experimental design used here to determine passive muscle stiffness. In other words, we cannot eliminate possibility that passive muscle stiffness would be influenced by the rs12722 polymorphism when measured in more stretched and therefore tensioned hamstring positions. Further studies will be required to clarify these points.

To the best of our knowledge, four studies have attempted to identify the association between the *COL5A1* rs12722 polymorphism and joint flexibility [10–12, 33]. Of these, three have indicated a potential association [10–12], which seems to be inconsistent with the present results. However, careful attention should be paid when interpreting the previous findings. For example, Collins et al. [11] and Brown et al. [10] included participants with a wide age range (although its effect was statistically adjusted). Especially in the latter study, a significant association between the *COL5A1* rs12722 polymorphism and SR ROM was observed in the old age (≥ 35 years) group but not in the young age group (< 35 years) or combined group (i.e., all participants) [10]. The present study examined only young populations (≤ 32 years) and found no association between the rs12722 polymorphism and joint flexibility. There remains a possibility that the influence of the rs12722 polymorphism on joint flexibility is age-dependent. Another possible explanation for not only this inconsistency regarding joint flexibility but also the aforementioned discrepancy in the association of the *COL5A1* rs12722 polymorphism with injury severity is the difference in participant ethnicity: Caucasian [10, 11, 18, 19] vs. East Asian. A recent meta-analysis indicated that the *COL5A1* rs12722 polymorphism was associated with tendon and ligament injuries in Caucasians but not in Asians [25]. The T allele frequency of the rs12722 polymorphism is lower in East Asian [0.220 in East Asian population of the 1000 Genomes [34], 0.162 in Japanese population of the Integrative Japanese Genome Variation Database [35]] than in Caucasian [0.585 in European population of the 1000 Genomes [34]] populations. If the optimum model in the association of the rs12722 polymorphism with joint flexibility and muscle injury is T-recessive or additive, the potential association may not be found due to the low frequency of the TT genotype in East Asian populations ( [33] and the present study). In the present study, however, joint ROMs and passive muscle stiffness in TT genotype carriers were comparable to those in TC + CC genotype carriers. All in all, these facts suggest that the *COL5A1* rs12722 polymorphism plays a minimal or negligible role in sports-related muscle injury risk.

A limitation of the present study is the cross-sectional design, which does not permit a mechanistic explanation. Thus, large-scale follow-up studies would be desirable to conclude whether the *COL5A1* rs12722 polymorphism is associated with muscle injury. Another limitation is that we have tested only the *COL5A1* rs12722 polymorphism. It is possible that other polymorphisms within the *COL5A1* as well as the genes for other types of collagen are associated with the joint flexibility, passive muscle stiffness, and muscle injury. Further investigations are warranted to identify these.

## Conclusions

The present study does not support a role for the *COL5A1* rs12722 polymorphism in sports-related muscle injury in a Japanese population. Furthermore, we provide evidence suggesting that the polymorphism is not related to passive muscle stiffness or joint flexibility.

## Supplementary information

**Additional file 1: Table S1.** Participant characteristics by COL5A1 genotype in men and women of Study 1.

## Data Availability

The datasets analyzed during the present study are available from the corresponding author on reasonable request.

## Abbreviations

ANCOVA: Analysis of covariance
BF: Biceps femoris long head
CI: Confidence intervals
COL5A1: Collagen type V α1 chain
OR: Odds ratios
PSLR: Passive straight-leg-raise
ROM: Range of motion
SM: Semimembranosus
SR: Sit-and-reach
ST: Semitendinosus
UTR: Untranslated region
